# Developing a socio-ecological model of dietary behaviour for people living with diabetes or high blood glucose levels in urban Nepal: A qualitative investigation

**DOI:** 10.1371/journal.pone.0214142

**Published:** 2019-03-25

**Authors:** Lizzie Caperon, Abriti Arjyal, Puja K. C., Jyoti Kuikel, James Newell, Remco Peters, Andrew Prestwich, Rebecca King

**Affiliations:** 1 Nuffield Centre for International Health and Development, Leeds Institute of Health Sciences, University of Leeds, Leeds, United Kingdom; 2 HERD International, Prasuti Griha Marg, Kathmandu, Nepal; 3 School of Psychology, University of Leeds, Leeds, United Kingdom; University of Botswana, BOTSWANA

## Abstract

Instances of non-communicable diseases such as diabetes are on the rise globally leading to greater morbidity and mortality, with the greatest burden in low and middle income countries [LMIC]. A major contributing factor to diabetes is unhealthy dietary behaviour. We conducted 38 semi structured interviews with patients, health professionals, policy-makers and researchers in Kathmandu, Nepal, to better understand the determinants of dietary behaviour amongst patients with diabetes and high blood glucose levels. We created a social ecological model which is specific to socio-cultural context with our findings with the aim of informing culturally appropriate dietary behaviour interventions for improving dietary behaviour. Our findings show that the most influential determinants of dietary behaviour include cultural practices (gender roles relating to cooking), social support (from family and friends), the political and physical environment (political will, healthy food availability) and individuals’ motivations and capabilities. Using these most influential determinants, we suggest potentially effective dietary interventions that could be implemented by policy makers. Our findings emphasise the importance of considering socio-cultural context in developing interventions and challenges one-size-fits-all approaches which are often encouraged by global guidelines. We demonstrate how multifaceted and multi layered models of behavioural influence can be used to develop policy and practice with the aim of reducing mortality and morbidity from diabetes.

## Introduction

Non-communicable diseases [NCDs] are a leading cause of death globally with 70% of global deaths due to NCDs in 2015. This figure is projected to increase significantly [1]. The burden of NCDs is highest in low- and middle-income countries [LMICs] where over three quarters of global NCD deaths [30.7 million] occurred in 2015 [1]. Diabetes is the fourth most common NCD after cardiovascular disease, cancer and respiratory diseases and is responsible for 1.6 million deaths globally [1]. As 80% of global diabetes deaths occur in low income countries [2] it is a disease which needs tackling in these settings. Low income South Asian countries, such as Nepal, have seen a particularly rapid increase in the prevalence of diabetes in the past two decades. South Asians are at increased risk of diabetes compared with other populations such as Caucasians [3–5].

Unhealthy diet (high in salt, sugar and fat and low fibre, fruits and vegetables) is a cause of diabetes [3, 6, 7]. A healthy lifestyle consisting of regular exercise, limited alcohol and no tobacco use and a healthy diet consisting of fruit and vegetables, less sugar, salt and saturated fats can contribute to the prevention of NCDs such as diabetes [8]. However, with the increasing availability and demand for unhealthy foods [9–13], encouraging healthy dietary behaviour is a growing global challenge. When we refer to healthy or unhealthy diet or eating we do so in relation to a healthy diet for everyone, not just for people with diabetes. We use the WHO advice for healthy diet but account for socio-cultural variation within this generic advice, for example, allowing adapting a generic recommendation for the consumption of pulses to be made specific to the lentils and dahl commonly consumed in Nepal [14]. Dietary behaviour can include not only the consumption of, but the preparation or acquisition of healthy food which affects the ability to eat healthily. Numerous interventions have been trialled in both LMICs and high income countries [HICs] to improve dietary behaviour. A recent review found that of 76 randomised controlled trials [RCTs], which aimed to improve dietary behaviour in LMICs, only 7 of these addressed diabetes. Very few studies [15–17] have considered the determinants of dietary behaviour in diabetes patients specifically and none have been conducted in LMICs. This study fills the gap in existing evidence by considering the determinants of dietary behaviour amongst people with diabetes or high blood glucose levels [HBGLs] in a specific LMIC context.

This study focuses on diabetes in Kathmandu, Nepal. Increasing urbanisation has been linked with increased incidence of diabetes in Nepal [18, 19]. Urban and rural prevalence of the disease are 8.1% and 1% respectively [20]. NCDs in general are a substantial problem in Nepal; they are estimated to account for 60% of deaths [21]. Determinants of NCDs have been found to be multiple and varied [22–25]. In Nepal specifically, the rates of diabetes are increasing; in 2015 there were 526,000 reported cases of diabetes, and many more reported cases of HBGLs. By 2017 this had risen to 657,200 reported cases [26]. The number of cases of diabetes in adults which are undiagnosed is predicted to be 323.7 per 1000 people [27].

Nepal, like many other LMICs, has great problems meeting the World Health Organisation [WHO] target of fruit and vegetable intake; 99% of the Nepalese population do not meet the recommended daily allocation of fruit and vegetable intake [28]. Policy is currently failing to effectively tackle the causes of NCDs such as unhealthy diet [29]. However, some government attempts have been made to address the problems of unhealthy diet such as the multi-sectoral action plan for the prevention of NCDs [2015–2020] [28]. Additionally, the package of essential non-communicable diseases [PEN] first trialled in several districts in in Nepal in 2015 attempts to improve diagnosis and prevention of NCDs such as diabetes by improving screening processes in primary health centres [30], though the evaluation of PEN is yet to be conducted on a large scale. A recent study of barriers to diabetes services for patients in Nepal concluded that additional financial and human resources were needed in the health sector in addition to initiatives to improve diabetes awareness and management [31]. Despite some recent policy developments by the Nepalese government, there is a need to develop new interventions designed to prevent the increase of diabetes.

We develop an ecological model to illustrate the influence of multiple determinants of behaviour and to improve understanding of these. Ecological models have incorporated multiple determinants into different levels of influence on behaviour (intrapersonal, interpersonal, organisational, community and public policy) and consider the interaction of behaviours across these different levels of influence, which leads to multi-level suggestions for interventions to effectively change behaviour [32–36]. Levels of influence used in models previously include individuals, groups and organisations, whereas this study considers dimensions of influence in the form of environments, such as the physical environment, and socio-cultural context [37]. We define culture as a shared system of meaning which is adaptive and always changing. Culture includes conscious and unconscious assumptions and helps individuals interpret their experiences[38]. Embedding cultural understanding in global health has been shown to be important in developing effective health strategies [39, 40]. However, culture has often been used as if it is static and a barrier to health improvement, often conflated with related concepts such as race and gender. To better consider the influence of culture on health, we do not treat it as an independent and time-independent categorical construct. Instead, because culture is also affected by, and affects, social contexts, we consider social and cultural context together. We consider social contexts to include environments made up of conditions which include socially acceptable standards and customs. These conditions are influenced by groups or social circles with whom individuals interact within the context. Therefore we have chosen to categorise *socio-cultural context*, drawing on elements of the socio-cultural psychological approach [41]. This approach emphasises that cultural factors such as language, art, social norms and social structures can play a significant role in the development of cognitive abilities and defining behaviours. We draw on this approach to consider factors such as social norms (informal understandings that govern the behaviour of members of society [42]), social structures (e.g. family, communities) and cultural practices/traditions (e.g. fasting, feasting) as playing an important role in behaviours. Socio-cultural context incorporates social factors (social relationships, social support) with cultural aspects (shared systems of meaning) to reflect values, norms, customs, influences and traditions. We use the term socio-cultural context as interchangeable with the term socio-cultural environment, the former forming an important part of our ecological model.

Environmental influences on dietary behaviour are increasingly discussed in the literature [43–45], one recent paper making clear different features of the environment which can influence dietary choices [46]. However, many studies focus on the obesity epidemic rather than dietary behaviour more generally, our focus being the latter. Some social ecological models in particular, see culture as important in interventions [47, 48]. We use our ecological model to better understand the research context in-depth and argue that one-size-fits-all thinking about intervention design is not appropriate. Though there is a plethora of global advice about healthy eating and diet, it is rarely contextually tailored to context [14, 49, 50]. We hope that our model can be used to aid the development of effective interventions to tackle risk factors associated with diabetes and other health challenges Our objectives are first, to develop an ecological model to expose the importance of broader influences on the dietary behaviour of people with diabetes and HBGLs in urban Nepal by understanding a research context in-depth, and second to investigate how thinking ecologically can lead to the development of a more tailored, contextually-appropriate and effective intervention design.

## Methods

### Qualitative study

#### Study design

Qualitative research was conducted in this study. A range of methods including focus groups and surveys have previously been used in other research to establish dietary determinants in HIC settings [17, 51–53], however, to the best of our knowledge, little research has used semi-structured interviews [SSIs] to establish dietary determinants in either HIC or LMIC settings. We used SSIs which is a method involving the compilation of a semi-structured interview guide which provided a clear set of instructions for interviewers, but with the opportunity for interviewers to explore emerging themes from the data in a flexible way. Our intention with using SSIs was to provide reliable, comparable qualitative data. Using SSIs, we aimed to produce socially useful knowledge using a pragmatic approach [54] whereby we oriented our study to address the problem of unhealthy diet to impact policy and practice. Conducting SSIs with patients and their families or partners allowed us to investigate the behaviours of people with diabetes and HBGLs at least three months after they were diagnosed with this condition so that there was enough time since diagnosis to explore changes in dietary behaviour. We were interested in understanding any behaviour change which patients had undertaken since diagnosis and in what form this change (or lack of change) had taken and why. To encourage discussion with patients we used participatory methods such as mapping their daily eating habits according to time of day [55–57], and mapping their access to people and places from their homes [58, 59], such as to health facilities or friends’ houses. We also used a Nepali calendar which listed cultural festivals and events as a prompt to encourage discussion of eating practices at different times of the year. In addition to patients and their partners, we interviewed health workers, researchers, policy makers and senior clinicians to better understand the context surrounding patients which influenced their dietary behaviours including an exploration of the existing dietary support available.

We purposively selected patients and their partners (n = 22) from one public hospital in Kathmandu, health workers (n = 9) from both public and private health sectors, policy makers (n = 2), researchers (n = 3) and senior clinicians (n = 2) from various institutions and public bodies. Participants were approached directly by the researchers either in a healthcare setting or via a phone call to their place of work. Low and low-middle income patients were chosen because; a) deaths from NCDs have been found to be highest in lower middle income groups [60] and b) we wanted to understand the challenges facing diabetes patients with limited resources and the majority of Nepal’s urban population fall into the categories of ‘poor’ (13%), ‘vulnerable’ (35%) or ‘middle class’ (47%) [61]. We categorised income groupings according to data in the Nepal Household Survey which found the average Nepali household to be earning 30,121 Nepali Rupees (NPR) per month [62] and taking advice from our Nepali colleagues at the health research company HERD International in Kathmandu about average incomes in Kathmandu. We classified low income as earning less than 50,000 NPR, low-middle income as earning 50–75,000 NPR, middle income as earning 75–100,000 NPR and high income as earning more than 100,000 NPR [62]. We purposively sampled people with diabetes and HBGLs to include people of different ages, genders, religions, ethnicities, income groups and time since diagnoses. To add richness to the data, we included some interviews conducted with both partners in a couple when one partner was diabetic or with HBGLs. We chose some couples where wives were the patient, and some where husbands were the patient to provide a variety of responses (See Table 1 for a breakdown of the patient characteristics). This methodology has been used successfully before [63–70], allowing partners to encourage each other to provide further information. Maximum variation sampling was used within the catchment of the area patients were recruited from [71]. Maximum variation was sought across age, gender, ethnicity, condition and religion to demonstrate important shared patterns which cut across the cases. We chose health workers from public and private health settings to gather an understanding of care in various settings. Additionally, we chose policy makers from key governmental departments involved in NCD policy, researchers involved in investigating NCDs in Kathmandu, and senior clinicians who were involved in implementing NCD policy in clinical settings (see Table 2). These stakeholders were selected through consultation with colleagues at HERD International allowing us to select individuals we thought most appropriate to interview under the projects aims and objectives.

**Table 1:** Characteristics of patients who participated in individual patient interviews (May–June 2017).

ID	Age	Gender	Partner (P) or single (S) interview	Income group *(Average earnings per month in Nepal Rupees)*	Ethnicity	Religion	Diagnosis	Time since diagnosis	Number of people in household
35	51	F	S	Rs. 50,000–75,000	Chhetry	Hindu	Diabetes	9 years	4
34	55	F	S	Less than Rs. 50,000	Sherpa	Buddhist	Diabetes	5 years	5
45	52	M	P	75,000–100,000	Brahmin	Hindu	Diabetes	2 years	5
44	33	M	S	50,000–75,000	Chhetry	Hindu	Diabetes	4 years	3
46	60	F	S	50,000–75,000	Brahmin	Christian	HBGLs	14 years	4
38	54	F	S	More than 1,00,000	Magar	Hindu	HBGLs	1 year	5
33	59	F	P	less than Rs. 50,000	Newar	Hindu	Diabetes	1.5 years	4
32	62	F	P	less than Rs. 50,000	Brahmin	Hindu	Diabetes	12 years	3
26	45	M	S	50,000–75,000	Chhetry	Hindu	Diabetes	5 years	4
68	51	M	S	More than 1,00,000	Brahmin	Hindu	HBGLs	2 months	3
73	38	F	S	More than 1,00,000	Brahmin	Hindu	Diabetes	4 years	5
75	35	F	S	less than Rs. 50,000	Chhetry	Hindu	Diabetes	11 years	5
1	46	M	S	Less than 50,000 NRs	Newar	Hindu	Diabetes	9 month	5
6	39	F	S	50,000–75,000 NRs	Chettri	Hindu	Diabetes	10 years	7
10	55	M	S	Less than 50,000 NRs	Rai	Hindu	Diabetes	9 years	6
8	49	M	P	Less than 50,000 NRs	Chettri	Hindu	Diabetes	13 years	4
11	56	F	P	Less than 50,000 NRs	Magar	Hindu	Diabetes	5 years	5
12	55	M	P	More than 1,00,000	Brahmin	Hindu	Diabetes	7 months	6
7	63	M	S	Less than 50,000 NRs	Chettri	Hindu	Diabetes	8 years	7
23	52	F	S	Less than 50,000 NRs	Newar	Hindu	HBGLs	2 years	4
82	57	M	S	More than 1,00,000	Brahmin	Hindu	HBGLs	10 months	4
83	44	M	S	Less than 50,000 NRs	Chettri	Hindu	HBGLs	3 months	5

**Table 2:** Characteristics of health workers, researchers, senior clinicians and policy makers who participated in individual interviews (May–June 2017).

ID	Type of Interviewee	Gender	Occupation
01HW	Health worker	Female	Dietician
02HW	Health worker	Female	Dietician
03HW	Health worker	Female	Dietician
04HW	Health worker	Female	Community health centre worker
05HW	Health worker	Female	Community health centre worker
06HW	Health worker	Female	Community health centre worker
07HW	Health worker	Female	Clinician
08HW	Health worker	Male	Clinician
09HW	Health worker	Male	Community health leader
01SH	Senior clinician	Male	Clinician
02SH	Researcher	Female	Researcher
03SH	Senior clinician	Female	Clinician
04SH	Policy-maker	Male	Government official
05SH	Policy-maker	Female	Government official
06SH	Researcher	Male	Researcher
07SH	Researcher	Male	Researcher

An iterative approach was taken when developing the SSI guides which consisted of open questions structured around topics derived from literature reviews of previous research about dietary behaviour amongst people with diabetes and using the COM-B framework [72]. The themes used for patients included: dietary change since diagnosis, current and previous eating habits, social aspects of eating (who does participant eat with), purchase of food (who makes these decisions in the household), mapping dietary habits to annual calendar and discussion of festivals, support received since diagnosis (including mapping of support activity) and support desired. The themes for health workers included: description of service provided for people with diabetes/HBGLs, communication with patients, dietary patterns observed amongst patients, perceptions of patient’s capacity to change their dietary behaviour, what motivates patients’ dietary behaviour, capacity of health workers to provide better support for patients and factors affecting health workers abilities to do this. The themes used to interview policy-makers/researchers included: aspects of their work which encourage healthy eating in people with diabetes/HBGLs, what has been effective in providing dietary support, barriers and obstacles to improve dietary behaviour amongst people with HBGLs/diabetes. Embedding COM-B in our themes allowed us to consider the factors affecting the **C**apability of the target population to eat healthily such as cooking skills or knowledge, factors affecting their **O**pportunity to enact such behaviours (affected by environmental influences) such as social or cultural pressures encouraging them to adopt particular dietary behaviours, and factors that influence their individual **M**otivation to have a healthy diet. COM-B was chosen to allow for the consideration of multiple components which may act on an individual and influence their behaviour and has been successfully applied in other contexts [73–75]. Two patient interviews were undertaken as pilots. These were transcribed from audio recordings first in Nepali, and then they were translated into English. Next, the research team reflected on the pilot interviews and revised and improved the interview guides (see S1 File and S2 File for examples of the interview guides used).

#### Data collection

Data collection was coordinated by HERD International in Kathmandu which specialises in health research and has worked on the topic of diabetes before, meaning they had awareness of the issues around diabetes care in Kathmandu. Interviews were conducted in the homes of patients (n = 22), in health care facilities (n = 9), government offices (n = 2) schools (n = 1) or in the HERD International offices in Kathmandu (n = 4). All locations provided privacy and quiet space to allow participants the opportunity to speak calmly and openly. Patient interviews were conducted by two Nepalese researchers from HERD International undertaking postgraduate training in health research methods [JK &PK]. Health worker and policy-maker interviews were undertaken by the lead researcher [LC], a PhD researcher with experience in qualitative research collection. When it was not possible to conduct health worker interviews in English, the lead researcher [LC] was assisted by Nepali researcher [JK &PK] acting as a translator. Interviews lasted for approximately one hour each. Health workers were not present during patient interviews to allow patients to speak freely. Reflective field notes were taken by researchers straight after the interviews in English to inform the subsequent interview. All interviews in Nepali were transcribed from audio recordings first in Nepali, and then they were translated into English. All interviews in English were transcribed from the audio recordings in English. In line with both UK and Nepali ethical requirements, information sheets and consent forms were provided to all participants. We conducted 38 interviews in total which was the maximum number we could pragmatically conduct in the time and budget constraints of the project.

#### Data analysis

Like the sampling and data collection process, the data analysis took an iterative form. Data was constantly analysed and refined as it was collected, with the majority of the data analysis conducted in-depth at the end of the study. Data was managed in NVivo and analysed using the Framework Approach [76]. This approach was pragmatic and selected to allow themes to emerge inductively from the data, and also to allow pre-determined objectives to lead to deductive analysis [54]. The interviews were first coded according to the main themes explored in the SSI interview guide [COM-B]. Following this it became clear that key themes were emerging from the data around environmental influences on behaviour (e.g. physical environment shown by access to particular food, socio-cultural environment shown by cultural influences on dietary behaviour). Therefore, a second round of coding took place which developed these emergent themes further and moved away from the COM-B framework and towards the themes we propose in our ecological model. In line with these emergent themes, three members of the research team (LC, RK, RP) independently coded a sample of three interviews and then through discussion reached a consensus on a coding frame including a priori and emergent codes. LC then coded the other interviews, discussing any amendments to the coding framework with RK and RP. The major themes appeared repeatedly in interviews and carried importance in the interview discussions. The ecological model we propose emerged from the classification and discussion of the key themes.

This study received ethical approval from:

1. Nepal Health Research Council, RamShah Path, Kathmandu, PO Box 7626, Nepal

2. Faculty of Medicine and Health Research Ethics Committee, University of Leeds, Worsley

Building, Leeds, LS2 9LJ

## Results and discussion

### Characteristics of the respondents

SSIs were held with 22 patients—11 males and 11 females, aged 33 to 63 (see Table 1). Of these 22, 6 were patients with HBGLs but not diabetes. The majority were Hindu, and some were Buddhist or Christian. The majority of patients were in the low income (n = 11) or low-middle income (n = 5) brackets, with 1 patient earning middle income and five with high income. Seven patients were interviewed with their partners. We tried conducting an interview with each partner separately, however found that the rapport and recall was noticeably better when both partners were interviewed together and were able to respond to comments the other made and this also showed the dynamics between them. SSIs were also conducted with 9 health workers and 7 policy makers/researchers/senior clinicians. We defined senior clinicians in this group as distinct from the health worker group as they were clinically trained individuals who had close links with policy makers and were active in government policy implementation or formation. They therefore offered a more overarching view on policy. Of the health workers, 7 were female and 2 were male, 2 were clinicians and 3 were dieticians. The majority of health workers were from the public sector (n = 7) with two from the private sector. Of the policy makers, 1 was female and 2 were male, of the researchers, 1 was female and 2 were male and of the clinicians 1 was male and 1 was female.

### Key themes emerging from the analysis

Our data analysis grouping of key codes into important themes revealed three levels of influence according to the scope and broad nature of their influence. The first theme was those factors which related to the environment around the individual; second were intermediate environments—political and physical; and finally the environment with the broadest influence was the socio-cultural environment. We discuss these four different themes; individual characteristics and behaviour, physical, political and socio-cultural. These are displayed in Table 3.

**Table 3:** Levels of themes emerging from the analysis.

Level of influence	Theme	Sub-code (where appropriate)	Code of specific influencing factor
**Individual**	**Individual environment**	Personal psychological capabilities	Individual capacity for motivation
			Individual capacity for change
**Intermediate**	**Physical environment**		Access to ‘outside’ food and fast food
			Consumption of food in the home
			Availability of healthy food and junk food
	**Political environment**		Government campaigns and policy
			Political will
**Higher/broader**	**Socio-cultural context**	Cultural practices	Culturally appropriate food
			Ethnic dietary practices
			Religious dietary practices, festivals and fasting rituals
		Social support	Support from family (household), friends, community
		Gender constructs	Socio-culturally constructed gender roles
			Female/male involvement in food and cooking

#### Individual behaviours

Within the personal environment are *personal psychological capabilities such as the internal capacity for*, *and motivation to change one’s behaviour*. These are the C and M of the COM-B framework [72]. Our data indicates a lack of *motivation* to eat more healthily:

*‘I even have things like sweets that I am not supposed to have*. *It does make an impact on me.’ Female Diabetic*

Predominantly we found that patients had not known about healthy eating before they were diagnosed with diabetes or HBGLs. Some patients found it hard to change their dietary habits even after receiving advice to eat a healthy diet following their diagnosis. Often controlling diet was hardest during festival periods, showing that their socio-cultural environment affected on personal behaviour:

*‘I can eat what I want for 1–2 days*. *After all Dashain [annual festival in Nepal] is a festival of only 4–5 days so I eat everything during Dashain.’ Male diabetic*

Those who did resist cultural norms were aware that they were not participating in these practices, and of the possible social consequences of not participating. Understanding such cultural nuances appears to be vital when considering individual motivations and abilities to change behaviours. There is tension between cultural determination and individual agency in relation to dietary behaviour as has been found in other research [77, 78]. Further research is needed into this tension, and the determinants within socio-cultural context and individual agency which affect ultimate decision-making. Our findings also corroborate other research as they suggest that knowledge and awareness alone is not sufficient to action behaviour change. Michie, van Stralen (72)’s behaviour change wheel attempts to tackle the problems associated with only providing dietary advice to patients by suggesting that interventions should trigger action (which effect opportunities, motivations and capabilities) rather than simply imparting knowledge about how to behave. Our findings suggest that such interventions should trigger action by incorporating socio-cultural context which influences behavioural decision-making. These are important considerations for developing an effective intervention.

#### Physical environment

The physical environment presented challenges regarding the acquisition and *availability of healthy foods*. Some patients who were aware of the impact of *unhealthy junk foods* on their health, stated that they found it difficult to eat outside due to the oily nature of ‘*outside’ food*, and the lack of healthy options for diabetic or HBGL patients. Several patients stated that they ate fast foods such as momos and chow mein outside of the home:

*‘I eat this [momo’s and chow mein] outside*. *No one can make this in home.’ Male diabetic*

This finding suggests that unhealthy fast foods are often not *served in the home* and therefore ‘outside’ and ‘inside’ eating represent different spheres of influence on dietary behaviour; the home being traditional, and outside it being a new and potentially exciting space for consuming unhealthy junk food. Such new and fashionable outside spaces, and a fashion around inhabiting them, is likely to be a reason why many people are now choosing to consume these tasty foods high in saturated fat and salt. This is corroborated by other research findings that eating ‘outside food’ in restaurants and outlets is a relatively recent phenomenon in Kathmandu, supporting evidence of a nutrition transition in Nepal [79–81]. These findings are consistent with other research which has shown that junk food availability and consumption has been increasing in Nepal [79–84]. Also, as junk food becomes more affordable, there have been recent increases in vegetable prices, making healthy foods harder for poorer households in Nepal to afford [85].

#### Political environment

The political environment had the potential to influence behaviour with *government campaigns or policies* and legislative moves. An example of this was the government campaign ‘My Year’ or ‘Mero Barsha’, launched after Nepalese new year 2017 which aimed to encourage self-motivation adherence to five commitments; stopping consumption of alcohol, tobacco or tobacco-related products; exercising regularly; eating healthy and nutritious foods; having routine check-ups; and watching the health of family members. One heath worker stated that the ‘Mero Barsha’ campaign had the potential to influence her patients, but until then had not been effectively disseminated:

*‘Mero Barsha has a lot of potential to encourage my patients to eat more healthy foods but so far my patients have not heard of it and I have not been told to promote it to them*.*’ Health worker*

Health workers we interviewed were not familiar with the government campaign to promote healthy lifestyle, though government officials stated that the campaign was active and should be widely known about. Here discrepancies were evident between the presence of and dissemination of campaigns to improve dietary behaviour. One government official, when asked about policies which could improve dietary behaviour in Nepal, showed willingness to implement a sugar tax in the country:

*‘We have been through series of discussions with other stakeholders and we have received positive feedback from them and most probably the government will do something about increasing the tax on the sugar and sugar-related products*.*’ Policy-maker*

The same policy-maker discussed government commitments to introduce a ban on junk food in schools. Evidently, *political will* is in place to bring about changes to improve the political environment surrounding patients in Nepal. However, this political will was not evidently translated into political action. Therefore, though potential existed to bring about positive change in policy, the opportunities for the political environment to influence dietary behaviour positively appeared limited. The area of political change and policy warrants further research and this investigation has been started by the lead author who has analysed NCD policy in Nepal in more detail, and makes recommendations for policy-makers on how to improve dietary support for patients with NCDs, specifically diabetes.

#### Socio-Cultural context

As outlined in the introduction we see socio-cultural context as incorporating social factors (social relationships, social support) with cultural aspects (ethnicity, religion) to reflect society’s values, customs, influences and traditions. Cultural practices greatly influenced patients’ views on whether they should adopt healthy eating practices. The cultural consumption of certain foods was central to dietary behaviours. White rice was evidently vital to Nepali identity and eating practices:

*‘We are Nepali and we should eat rice. I do not care if you give me that list [from the doctor of foods which diabetic patients should/should not eat] or not but I will not follow it*.*’ Male diabetic*

Here it is important to consider why the participant feels he should eat rice. It was a commonly held cultural belief amongst participants that rice is a necessary part of every meal and it made them feel full. A meal without rice was seen as no meal at all. Rice is a filling food and therefore made participants feel full after eating it. It was clear that rice is heavily laden with cultural significance in Nepal, and consumption of it is central to being part of Nepali culture. The tern ‘bhat’ for rice, was used by many participants to mean ‘meal’, and ‘mam’ was a term used by participants for the rice that is fed to a baby, illustrating its embedded nature in culture from infancy. Patients found it difficult to break the cultural code of eating white rice and replace it with alternative carbohydrates such as roti. White rice ranks very high in the glycemic index which means it can cause a spike in blood sugar levels and consumption of it has been linked to diabetes [86]. For these reasons patients with diabetes or HBGLs are warned against consuming large amounts of white rice.

Despite the challenge involved in eating meals without rice, many patients told us they had managed to reduce their white rice intake to improve their health:

*‘I eat less rice now because I know it will make me sick*. *I try to eat roti instead.’ Female diabetic*

To aid this transition, some dieticians advised their patients that they could still eat small amounts of white rice as part of their diet. This allowed them to retain some of the cultural practice which was so central to their identity, whilst still maintaining a healthy diet. These findings are supported by the literature which has found that white rice is predominantly seen as the most filling and healthiest food to consume, an essential component to the Nepalese diet [83, 87]. Meals without rice are often seen as incomplete and a large helping of rice is seen as essential for health and well-being. Wholegrains like millet and buckwheat are not seen as good enough compared with rice [88]. The cultural significance of *culturally appropriate foods* such as rice and its complex meaning must be explored within the context of diabetes and healthy eating behaviour.

In addition to the importance of rice in Nepalese diets, in certain *ethnic groups different eating practices were important* due to varying ethnic and cultural traditions and rituals. Fasting for long periods is harmful to people with diabetes and HBGLs who need to eat regularly to regulate their blood sugar levels [89], however fasting can be a vitally important cultural practice in Nepal. Eating practices around festival times were particularly challenging. One patient described a *religious festival* which was observed by Hindu women called Teej, in which she did not eat food to honour her husband. She described how she felt like something was wrong when she tried fasting:

*‘I stopped fasting because I was told it was bad for me*. *But when I’m not fasting I don’t feel good and I feel like something is wrong…I’ve decided to fast again to feel better.’ Female patient, HBGLs*

Religious festivals and closely linked to ethnic dietary traditions. Our patients reflected the rich diversity of ethnicities in Kathmandu. Patients from the ethnic groups Newari and Chettri, both of which follow the Hindu religion, demonstrated particularly distinct ethnic dietary traditions throughout the year, often linked to festivals. Newari are the second largest ethnic group in Kathmandu (after Brahmin), making up approximately 22% of the population and Chettri make up 20% of the population [90]. During discussion of one of many festivals the Newari people celebrate, one patient stated:

*‘There was Sithi [Newari festival]*. *Then we cook bara [spiced lentil patties], varieties of potato and black eye bean curry. The lentils are soaked in water a day before and the next day the covers are peeled off, the lentils are ground and breads are made out of them. They are cooked in oil …The potato is boiled and then deep fried…I eat little bit of everything.’ Female diabetic [Newar]*

This discussion indicates the dietary practices followed by members of the Newari group. These include food preparation in a particular way for certain Newar festivals, including deep frying food which is an unhealthy dietary behaviour and could worsen her diabetes. However, the participant was compelled to eat this food due to it being part of an important ethnic practice. These traditions differed from patients from other groups such as Chettri, who have different dietary traditions surrounding their ethnic festivals. For example, even common foods, such as chapatti, were made differently by different ethnic groups:

*‘Our [chettri] chapatti is made up of Gyan chakki atta [all-purpose type of flour used to make chapatti]*. *The doctor has suggested me not to eat anything made from all-purpose flour, which is why I eat only wheat flour now.’ Male diabetic [Chettri]’*

This patient has demonstrated that he has been able to change an ethnic dietary practice to eat healthier flour as his doctor has recommended. However, as demonstrated by our Newari patient, and others interviewed, many found it difficult to make such changes, cultural pressures around ethnic practices being strong. Interventions could strongly benefit from being sensitive to cultural pressures to adopt certain dietary behaviours and could consider transmitting messages about healthy dietary behaviour through prominent cultural leaders. These findings are consistent with other research which suggests that culturally defined eating patterns affected behaviours [91]. Other research has found that patients still take part in fasting due to cultural pressure to do so [92–97] and religion has been explored as a determinant of dietary behaviour in other research [92–97]. Religious festivals often lead to eating large amounts of unhealthy, culturally defined foods in celebration [92–97]. This illustrates the importance of understanding the varied religious and ethnic cultures in Kathmandu and how they create, form and reinforce culture. Such cultures vary between communities and households making it important to tailor interventions to incorporate this understanding. Furthermore, acknowledging the importance of these traditions does not mean that they cannot be adapted to improve health outcomes to ensure they still meet the same goals, in the case of Teej, honouring the husband, though not to the detriment of the wife. Such cultural adaptation has been demonstrated in LMIC contexts previously. An example of this was changing a cultural practice which had negative health effects on babies in Kenya to find an alternative way of fulfilling this cultural tradition so that it had much better health outcomes [98].

#### Social support

*Social support* appeared to be an important determinant of the dietary behaviour of patients we interviewed. Patients very often attended medical appointments with family members showing the importance of family. Additionally, many people felt the pressure of caring for their families as well as their own health; with some patients, this acted as a motivating factor in ensuring adherence to control their diet and limit the intake of foods such as deep fried or oily foods. One dietician asked patients to act as role models for their children, explaining that now the patient was diabetic, their children were already at risk. One patient indicated he was consciousness of taking care of his family now he had diabetes. He demonstrated this by ensuring they all eat healthy food:

*‘Moderator*: *That means you all eat the same food?*

*Participant: Yes it will be the same because I am conscious that maybe my children will also have sugar [south Asian term for diabetes] because I have it*. *So I have given them knowledge so that they will not suffer from sugar for a long time and they are also aware of their eating habits.’ Male Diabetic*

This supportive behaviour shows the powerful positive influence *social support from family* in the social environment surrounding patients to bring about behaviour change. Patients appeared to receive a lot of support from their social relationships. These relationships were found with family and friends and within communities. Our findings are corroborated by other research which has found that family responsibilities are culturally very important in Nepal [83]. Family bonds and responsibilities, often multi-generational in nature are vastly important in Nepal, and these traditional structures often act as key drivers in behaviours [99]. The connection between showing love through presenting loved ones with food has been previously explored [100]. The power of social influences on eating behaviours have been found in high income country contexts [101–105], particularly the influence of family and friends [106]. Evidently social factors in the socio-cultural environment can affect dietary behaviours.

#### Gender constructs and roles

*Socio-culturally constructed gender roles* were another important influence on determining dietary behaviours and were discussed amongst all participants interviewed. We found gender roles to be entrenched in Nepalese society. Patients we interviewed told us that *male/female involvement in food and cooking* varied. One example of female involvement in food and cooking, referred to by several participants, was the role the daughter-in-law played in providing food for the household. Daughters-in-law played an important role in households by cooking and providing food for their husband and his family, showing a continuation of cultural traditions even in a modernised urban environment. However, daughters-in-law did not always prepare healthy food:

*‘Our daughter-in-law cooks all types of meat like a roast and we eat that*. *I just eat a little because I get scared because that is very oily.’ Female Diabetic*

The role of preparing healthy food, that is for example, vegetables cooked with small amounts of oil, often sits in the hands of specific female members of the household. There was evidence in some households that women had been made aware of the importance of cooking healthy food for diabetic family members, and that this had a positive effect on the dietary behaviour of the diabetes patient. Sometimes the male in the home helped to cook, particularly during times when the female cook was menstruating (usually if there was no other female who could cook in the home, which there often was not):

*‘I was menstruating on that day so I did not have to participate in any of the work*. *They gave me rice, meat, vegetables and pickles.’ Female Diabetic*

This links to the Nepalese cultural practice of ensuring menstruating women do not enter the kitchen so as not to pollute or dirty the food. This poses a real challenge for diabetic women who are not permitted to enter the kitchen when they are menstruating, yet they do not feel that they can ask for special food which is suitable for them. Traditional cultural practices are influential practices in daily life. Other studies into household behaviour corroborate our findings as they have shown that women are key gatekeepers in influencing household dietary behaviour and educating them about healthy eating practices has great potential to improve the dietary behaviour of entire households [83, 94, 107]. This is particularly the case because men are often found to have less healthy dietary practices than women in Nepal and elsewhere [108, 109]. Additionally, daughters-in-law were often tasked with cooking for the entire household, and take the last position in household serving order [88, 107, 110–112] with women often eating only once men have finished [88]. This can be down to a range of factors including power dynamics with their mother-in-law [113] This has implications for both men and women with diabetes, for example women in the household may consume unhealthier food, or carry a burden of caring for other members of the household, potentially causing higher levels of strain both physically and mentally. Understanding gender roles within Nepali households is therefore vital to assessing how interventions might effectively influence behaviours within the household.

### An ecological model for dietary behaviour

**Fig 1:**
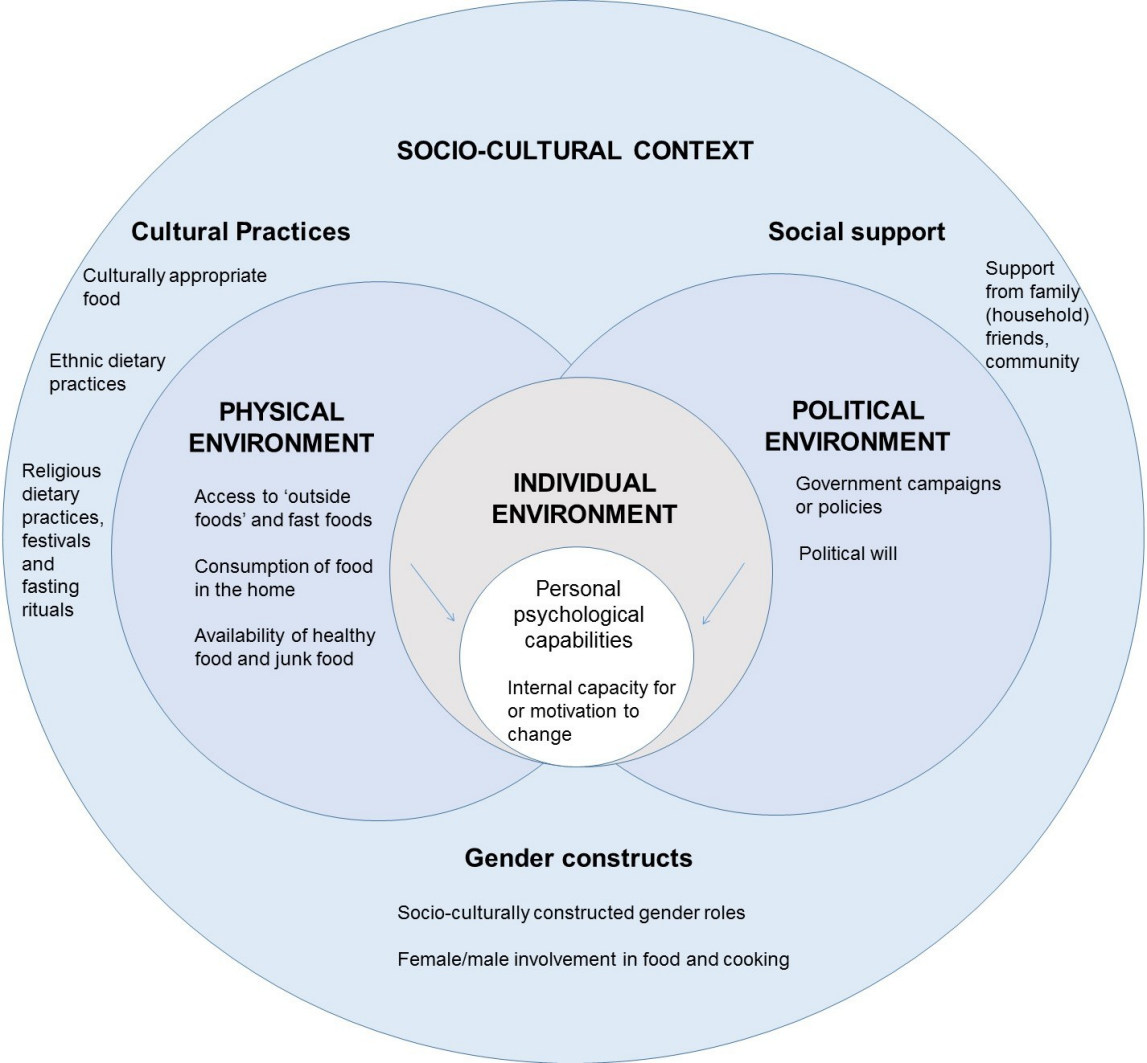
Ecological Model of determinants of dietary behaviour in patients with diabetes and HBGLs in Kathmandu.

Our findings uncover several layers of influence which determine dietary behaviours of people living with diabetes or HBGLs. Our ecological model (Fig 1) demonstrates these multiple layers. Though influenced by previous ecological models [34, 114, 115], our model’s value is in its ability to categorise the determinants of dietary behaviour amongst a population with a specific NCD and placing the socio-cultural context as the dominant influencing environment on behaviour. These findings align with other research which has shown cultural specificity and sensitivity to be key in implementing health interventions [116–118]. Our findings corroborate the need for more culturally sensitive and adapted approaches to health interventions. Existing ecological models from HIC contexts do not consider socio-cultural context as such an important determinant of behaviour [34, 115, 119–121], and would therefore struggle to account for findings from our study which indicate the importance of the socio-cultural context on influencing dietary behaviour.

The model is constructed of four interacting environments, these are; socio-cultural, political, physical and personal environment (individual characteristics and behaviours). Behaviour specific models are useful and often appear in an ‘onion’ structure to represent multiple measures of influence [115, 121, 122] and ours roughly follows this structure. The overarching environmental influence is socio-cultural context which was dominant in determining behaviours in various ways, for example cultural practices such as fasting, ethnically defined dietary traditions and the associations around rice were powerful forces in determining behaviours. Intertwined in these cultural practices are religious practices such as eating certain foods during the festivals which dominate the structure of the majority of the Nepalese calendar. Gender constructs also fall within the socio-cultural environment; these describe the way in which the gender norms of society influence behaviour. Socio-cultural environment is not only dominant, but it intersects with all of the other layers of influence, for example the impact of cultural festivals on personal motivations in dietary choices. Social aspects such as social support from family and friends of the socio-cultural context were influential in determining behaviour. Socio-cultural factors (social support, gender roles in cooking) can be linked by structures such as households and communities, both of which vary in terms of religious, ethnic and regional differences. Underneath the overarching socio-cultural environment there are two environments at the same level of influence as each other because our findings suggest they exert similar amounts of influence on behaviours; political and physical. These environments were not at prevalent in affecting behaviours are socio-cultural context, however they were of overarching influence over the individual. These two environments (physical and political) determined individual behaviour in varying and interacting ways. The physical environment defined aspects such as a patient’s distance to health services and opportunity to acquire desired foods. At the central level of influence in our model is the individual environment. This involves behaviours attached to the individual. Within the individual environment are personal psychological capabilities such as the internal capacity for, and motivation to change one’s behaviour. These are the C and M of the COM-B framework [123]. When classifying opportunities for the individuals to change their behaviours (the O of the COM-B framework), these span the entire ecological model, with many forces working on the individual to influence their behaviours. Here limitations lie with the way that the COM-B framework marries up with our ecological model. Though we started with COM-B when designing our interview guides, the data showed that COM-B was not adequate to fully structure our findings. Whilst it was useful to some extent, to classify individual behaviours within the ‘personal environment’ using COM-B (capabilities and motivations) as discussed, involved placing all external forces on the individual into the broad category of ‘opportunities’. This over-simplified the multi-faceted nature of these opportunities which the ecological model splits into multiple environments of influence (e.g. physical, socio-cultural). By thinking about these factors within their environmental contexts, we can begin to understand the collective influence of the environment on the individual’s capability, opportunity and motivation together. Our data therefore suggests interventions may be effective if they are collective, and potentially community-based (allowing for contextual variability between communities) and combine socio-cultural, physical, political and individual environments.

Our model differs from other ecological models as it places the socio-cultural environment around all other environments as the overarching influencing environment on dietary behaviour. Other models place less emphasis on the socio-cultural environment, for example, some place macro forces [34, 119] or government and industry policies [115, 120] as the overarching (outer) influence. Others acknowledge the influence of culture but see it as secondary to community and policy [121]. Furthermore, other models of behaviour have not proposed the socio-cultural environment as the overarching environment, as ours does; they instead see social, intrapersonal and physical environments as equally influential on behaviours [114]. Alternatively, modelling by Short and Mollborn [124] stress the importance of looking at social determinants within the complex systems which operate around an individual. Sallis, Cervero [115] come closest to our model by acknowledging the intersecting nature of the social cultural environment on others in relation to determinants of active living in the USA; however, they place policy as the overarching influence on behaviour and do not treat the social cultural environment as the most powerful environment as our model does. The questioning of individual-focused interventions and exploration of the role of structures outside the individual on influencing behaviour has been well established previously [125]. Our model stresses the importance of gender within socio-cultural context, another model by Cislaghi and Heise [126] places gender and power as the most influential factors over individual, social, material and institutional environments. Whilst this is a valuable interpretation, and our findings concur that gender is an important influencer, we prefer to place gender as a key part of the wider socio-cultural context as socio-cultural context dictates the manner in which gender manifests as an influence. Our model builds on this thinking further to advance socio-ecological thinking by proposing that the socio-cultural environment is the most overarching influence on dietary behaviours in an LMIC context and interventions should consider this influence on behaviour rather than developing interventions which only consider the individual without the influence of wider environments on their behaviour. However, often cultural considerations are not prioritised in public health interventions in LMICs where western or HIC constructed theories and methodologies have often been seen as easily transferable [127]. We believe that as our model differs from other existing models adds to the existing literature by providing a valuable insight which cannot be gleaned from models based on HICs (for example [34]).

Our study is not without limitations. We only recruited patients who attended one public hospital in Kathmandu and who were diagnosed and receiving treatment for their condition. Patients who attended other health facilities, and those who were not actively seeking treatment, would also have been valuable to interview to gather a broader understanding of treatment at different health facilities, and the challenges facing those patients who have had limited treatment. We were only able to interview a limited number of policy-makers, researchers and health workers due to budget and time constraints. Furthermore, we acknowledge that health workers in the informal private sector including unqualified practitioners play an important role in care provision in Nepal and could influence dietary behaviour. These people were not interviewed in our study, though some patients referred to the influence of traditional healers, and we acknowledge this as a limitation. Additionally, though our ecological model shows multiple levels of interaction and multiple variables of interaction across and within these levels, we are aware that sometimes ecological models do not adequately specify the most important hypothesised influences or provide enough information about how the broader levels of influence interact or the interaction of variables across levels, such as Matsudo, Matsudo [114] who do not consider an overarching environmental influence and the interactions between variables. One way this could be addressed is with further research using our model on a multi-level analysis to evaluate and examine the impact of interventions on outcomes at multiple levels [33]. Considering in more detail the different levels, such as for example culture within the health care system, is also important for more in depth understanding on how these components play a part in the whole multi-level model. Our model is also not exhaustive, it would be difficult to capture all determinants of behaviour within the environments, and we are aware others which were not discussed by our participants, for example body image or the impact of advertising [128] which can also influence dietary behaviours. Also, we are aware of limitations with the way we categorised our sample according to socio-economic position. We are aware that social positions in south Asian settings can be complex and income levels are only one of many several markers of socio-economic position (other non-income markers include caste groupings and influence positions). However, given the difficulties of measuring socio-economic position, we have limited our focus to income groupings. Similarly, we are aware that the use of the terms ‘healthy’ and ‘unhealthy’ diet are terms which can be open to cultural interpretation. Though as this study indicates, culturally appropriate foods are contextually specific and vary with socio-cultural context, we have defined healthy/unhealthy diet in alignment with WHO advice on healthy diet while accounting for socio-cultural variation within this generic advice (e.g. the importance of rice—ideally brown rice for people with or at risk of diabetes—as a carbohydrate staple food in Nepal) [14]. Finally, we are aware that we have discussed the political environment impact on behaviours only from the perspective of policy-makers and researchers as there was no mention of the impact of policy by the patients themselves. We acknowledge this as a limitation because policy was not discussed from a patient perspective. We believe, however, that given the constraints of this study, reflections from policy-makers and health workers on political issues (e.g. about sugar tax and government campaigns) were valuable in identifying policies which held the potential to improve dietary behaviour.

### Incorporating key determinants into interventions

The data suggests that the most effective interventions will be developed with full consideration of the various environmental determinants of behaviour in a multi-layered way considering multiple dimensions or environments rather than levels of influence. This does present a challenge for policy-making which follows a one-size-fits-all approach advocated by global guidelines [14, 49, 50, 129, 130]. Instead, this study advocates the importance of using global advice as a starting point, but then adapting it to the socio-cultural context using a multi-layered approach considering multiple environments (physical, socio-cultural, political, individual). An example of such a multi-layered approach might be to train family members to cook healthy, culturally appropriate foods in the home, engaging both men and women in household food preparation. Culturally constructed gender roles play a significant role due to the importance of male/female division in food preparation. This indicated the continuation of cultural traditions even in a modernised urban environment [88, 107, 110–112]. Increasing numbers of women are working outside the home, their changing role in society means that they now often have to work inside (domestically–cooking food etc.) and outside (paid employment) the home. This places increasing strain on women who are traditionally the members of the household who cook [83, 88, 131, 132], which could lead to less time being spent on food preparation in the home, and more convenience foods being consumed [131]. Socio-culturally appropriate and compelling interventions which draw on social bonds and cultural nuances and aim to equally distribute responsibility for healthy food preparation in the home hold the potential to improve dietary behaviour in households.

An alternative multi-layered approach which considers the physical, political, socio-cultural environments may be to harness the power of community spaces such as community health centres and the promised health promotion centres, as well as other spaces which act as hubs for the community, such as recreation facilities or parks. The power of the communities, which are themselves laden with multiple and intersecting cultural, ethnic and religious elements, has great potential for the development of effective interventions. For this to happen communities must be understood, listened to and then harnessed and utilised effectively to bring about change. Further examples of community-led interventions have been community camps discussed by our participants which disseminate healthy dietary advice to the community by using the structures in communities, such as mothers groups or Ward committees to implement contextually-appropriate change [83, 133, 134]. Interventions should not only be contextually-appropriate but also culturally compelling [48], by fully engaging people in interactive behaviours which link to cultural practices and beliefs which are entrenched within their identities and therefore are deeply meaningful to them. For this to happen, context and culture and their relationship to the individual must be deeply understood. If patients had more and regular contact points with advice-givers in a community setting accessible to them, where they could receive regular reminders about healthy eating habits, they might be more able to consistently change their behaviour long term, rather than relying on short term quick fix measures which do not properly consider the communities or the importance of long-term sustainable health care interventions.

Our data illustrated the importance of many ethnic and cultural practices to people in Nepal. The core of these cultural traditions, that is, the message they focus upon, such as in the case of Teej, honouring the husband, can be preserved but the way of conducting these traditions changed so they are not harmful to the woman if she is diabetic. This can also be addressed considering the practice of excluding menstruating women from the kitchen. Examples of how these practices could be adapted must be developed by the communities and individuals themselves. As explored, these might involve teaching the husband or other family members healthy cooking skills so that they are able to prepare healthy foods for his diabetic wife without her needing to enter the kitchen. Such cultural adaptation has been demonstrated in LMIC contexts previously [98] and holds great potential for improving health outcomes whilst ensuring the preservation of core cultural beliefs and rituals.

Our model provides the basis to develop, test and evaluate interventions. Whether interventions based on our model are effective or not would provide further validation to the model. To provide a valid intervention test, however, interventions would need to be developed in close alignment with our model. Often interventions are not developed with close alignment to models despite purporting to do so [135–137]. Awareness of these issues should be considered when taking our model forward for testing interventions.

## Conclusion

Our results suggest that socio-cultural context is paramount in underlining, defining and influencing dietary behaviours in Kathmandu. Some participants had demonstrated positive dietary changes since diagnosis to limit culturally normal foods such as rice, which they had since learned were unhealthy if consumed in large amounts. However, the challenge of improving dietary behaviours amongst the majority of people with HBGLs and diabetes, and those at risk of getting the disease, remains serious in Kathmandu. To tackle this issue, an overarching understanding of the socio-cultural nuances and multiple socio-ecological environments can be used to propose interventions which are culturally compelling by engaging communities, households and individuals in an adaptable way which are flexible to the context and protect cultural beliefs whilst leading to positive health outcomes. The possibility of adapting cultural traditions through consultation with communities about compelling ways to maintain core beliefs and rituals yet improve health outcomes by altering aspects of rituals is clear and should be further explored. This study has challenged the one-size-fits-all approach to tackling NCDs, and instead highlights the value of investigating contexts in-depth to gather a detailed understanding of the determinants of specific behaviours. Our ecological model the first one of its kind to understand dietary behaviour in people with diabetes or HBGLs in an LMIC with a focus on socio-cultural context. We believe that our model has applicability to other contexts and other conditions (such as other NCDs like cancer and cardiovascular disease). We propose tailored, contextually-appropriate intervention design which adapts global guidance to a specific context. Our model’s robustness and transferability requires testing in other contexts taking into account the fluidity of changing environments. Our model provides the basis to develop, test and evaluate interventions. Whether interventions based on the model are effective or not would provide further validation to the model. In this study we have demonstrated the potential of investigating research contexts ecologically to improve intervention design to effectively tackle health problems such as NCDs.

## Supporting information

S1 File Interview guide: Patients (and their partners). (PDF)

S2 File Interview guide: Health workers. (PDF)
