# Supplementary material for: Developing a socio-ecological model of dietary behaviour for people living with diabetes or high blood glucose levels in urban Nepal: A qualitative investigation
Source: PLoS One. 2019 Mar 25;14(3):e0214142. doi: 10.1371/journal.pone.0214142 (PMC6433239; doi:10.1371/journal.pone.0214142)
Supplement: S1 File — (PDF) [file pone.0214142.s001.pdf]

Supporting Information 1: Interview guide – Patients (and their partners)

Record the following information about the participant:

Name and ID number	
Age	
Highest level of education	
Socio-economic group	Less than 50,000 NRS<br>50,000 – 75,000 NRS<br>75,000- 100,000 NRS<br>More than 100,000 NRS
Ethnicity	
Religion	
Working	Yes/No
Occupation/previous occupation	
Diagnosis	
Time since diagnosis (months or years)	
Other medical conditions	
How many people live with you most of the time? Who?	

1. I would like to ask you about when you were told you had diabetes/high blood glucose levels. What you knew about diabetes/high blood glucose levels before you were diagnosed?

Note to interviewer: If interviewing a partner of a patient separately, ask the above about their partners diagnosis

2. I would like to ask you about your eating habits and how/if these have changed since you were told you had diabetes/high blood glucose levels

Note to interviewer: If interviewing a partner of a patient separately, ask the following about their own eating habits (not their diagnosed partner). If their eating habits have remained the same, only use one template.

Exercise 1:

Provide a daily routine template. Ask the participant think about their diet BEFORE DIAGNOSIS. Ask them to write on the template (for an average day):

- What did they eat and drink when they got up (if they say they drank tea, did they have sugar in their tea)

- What did they eat and drink during the day, and where (at work, at home etc.)

- What they ate in the evening

Snacks

Now ask the participant to if they ate between meals, if they did, write down when they would eat and what they snacked on. Ask them where they got these snacks from and why they snack on them?

Sugary/sweet things

Ask the participant to write down if they ate sweet/sugary things, if so, how often, what kind of sweet/sugary things and where are they from?

Fruits and vegetables

Ask the participant to write down if they ate fruit and vegetables, why they ate certain fruit and vegetables (probe for pesticide use). Ask them how their vegetables were cooked?

Meat

Ask the participant to write down if they ate meat, if so, how often, what kind of meat and how was it cooked?

Rice or noodles

Ask the participant to write down if/when they ate rice, and how much rice they would eat during one meal?

Drinks

What drinks did you normally have? How much of each drink did you have? Where did you get these from?

Ask the participant if they are happy with what they have written, or if they would like to add anything

Exercise 2:

Provide another daily routine template. Now ask the participant to think about their diet SINCE DIAGNOSIS. Ask them to write on the template (for an average day):

- What they eat and drink when they get up (if they say they drink tea, do they have sugar in their tea)

- What they eat and drink during the day, and where they are (at work, at home etc.)

- What they eat and drink in the evening

Snacks

Now ask the participant to if they eat between meals, if they do, write down when they eat and what they snack on. Ask them where they get these snacks from and why they snack on them?

Sugary/sweet things

Now ask the participant to write down if they eat sweet/sugary things, if so, how often, what kind of sweet/sugary things and where are they from?

Fruit and vegetables

Now ask the participant to write down if they eat fruit and vegetables, why they eat certain fruit and vegetables (probe for pesticide use). Ask them how their vegetables are cooked (probe for fried/steamed, use of salt or oil)?

Meat

Now ask the participant to write down if they eat meat, if so, how often, what kind of meat and how is it cooked?

Rice

Ask the participant to write down if/when they eat rice, and how much rice they eat during one meal?

Drinks

What drinks do they normally have? How much of each drink do you have? Where do they get these from?

Ask the participant if they are happy with what they have written, or if they would like to add anything

Exercise 3: [Optional exercise with food models]

Ask the participants to arrange the food models according to what proportions of each food they think represents a healthy meal. [Take a photo of this model after the interview]

Ask the participant to describe the model they have made up.

[Using the drawings as visual aids ask the following]

Probes:

- How do you think your eating habits have changed since you (or your partner) found out you had high glucose levels/diabetes?
- Why did you stop/start eating the foods you eat/ate?
- [Compare the two diagrams] Which, if any, unhealthy foods have you found it hard to give up? Why do you find it hard to stop eating these foods? How much of these foods do you still eat?
- How you think you can still improve your eating habits? What would help you improve your eating habits?

3. I would like to ask you more about what eating is like for you. Describe the situation when you eat. How do you eat (with family, alone, one big meal, two big meals)? How would you like to eat your meals?

- Who decides what you eat in your household?
- Who prepares your food?
- How is their food prepared, e.g. with oil (what kind of oil?), spices, fried or boiled

4. I would like to ask you how you buy your food.

Where do you buy your food and who decides what food to buy?

If you could, would you buy different foods? Which foods would you buy?

5. I would like to ask you how culture and religion effects your eating habits

Exercise 4:

Provide a year calendar with months in Nepali and a pen for the participant.

Ask them to indicate if they observe any festivals/fasts during the year and about any weddings/funerals/other large events they have taken part in/will take part in in the next year, ask them to map these on the calendar.

If the participant provides any events which they observe ask them to indicate:

- why they observe these

- what foods they eat at each event

- Since their diagnosis have they changed what they eat at these events, if so, how do they feel about that? What do their family and friends think about them eating differently?

- if there are any fasting periods throughout the year

- if they fast at any point during the year

- If they don't fast now because of their diagnosis, how does that make them feel

[if interviewing partner, ask if their partner eats different foods to them and why/why not]

6. I would like to ask you about the support you have received since you were told you had diabetes/high blood glucose levels

[If interviewing partner of a diagnosed patient separately, ask them to map the support they AND their partner have received]

Exercise 5:

Provide the participant with a large sheet of paper and a pen, and some post-it notes.

Ask them to place in the middle of the sheet of paper a post-it note or mark indicating where they live.

What has helped you manage your diabetes/pre-diabetes?

What has made it hard to manage your diabetes/pre-diabetes?

House

Then ask them if anyone in their home helps them to eat more healthily. Ask them to indicate how much support they get from home by putting more [post-it notes]. Who does this support come from? [Probe: Support from partner or other family members, or ask if other family members actually make it harder for them to eat more healthily]

Individuals

If partner is present at interview ask:

Do you support your partner is to eat more healthily?

For patient [if they have a partner]

Does your partner support you? How/how not?

Do you visit the houses of other family and friends? If so, mark these on the map. Do they know about your diagnosis? If so, do they make special preparations for you?

Who has helped you since your diagnosis?

Places

Where did they go for diagnosis and then treatment? Do they still go there?

What has helped OR made things hard for them since their diagnosis?

PROBES:

- Distance to places they get drugs like pharmacy?

- Distance to places where they get food?

- Hospital, community clinic, food place, medicine shop (any), workplace, pharmacy, community health events?

- What support is received? How often is this support received? How useful is this support?

Ask the patient if they would like to add anything else?

7. I would like to ask you about how you get on with the people who provide you with support for your condition. Who provides you with support? How do you get on with them, do you respect them? Who do you prefer to get support from? Does gender, age or profession (Doctors/nutritionists/family, colleagues, friends) affect how you get on with the people who provide you with support?

8. I would like to ask you about what support you would like to receive for your diabetes/high blood glucose levels. Allow patient to answer unprompted.

- How likely would you be to attend a community event where you could learn more about healthy eating? Why?

- How likely would you be to attend nutrition education classes at hospital once per month? If not at hospital, would you be likely to attend classes if they were held in your community? Why/why not? Which would be easiest? [5.3 Information about social and environmental consequences, 11.2 Reduce Negative emotions]
- How willing would you be to take your partner *[or for partner if interviewed – go with your partner]* to nutrition education events to learn how to eat more healthily? [3.3 Social Support (emotional)
- How likely would you be to listen to advice from a family member or friend who had had training on how to eat healthily?
- How willing would you be in taking part in a program which allows your eating habits to be recorded (e.g. once per month or week) in order to help you eat more healthily? [2.4/2.3 Self monitoring of behaviour]
- How likely would you be to take a lunchbox into work so you can eat a meal during the day? Why/why not? [1.4 Action Planning]

  Probe: What issues would exist if you took a lunchbox into work? Social stigma about different types of food?

- How helpful would you find a reminder to eat certain foods daily or weekly help you to improve your dietary behaviour? [1.4 Action planning, 7.1 Prompts and Cues]

  Probe for whether the participant has a mobile phone and would respond well to a text message reminder to eat healthily

Is there any support you would like to receive for your diabetes which you don't already receive?

Thank you, do you have any questions?
