# Supplementary material for: Developing a socio-ecological model of dietary behaviour for people living with diabetes or high blood glucose levels in urban Nepal: A qualitative investigation
Source: PLoS One. 2019 Mar 25;14(3):e0214142. doi: 10.1371/journal.pone.0214142 (PMC6433239; doi:10.1371/journal.pone.0214142)
Supplement: S2 File — (PDF) [file pone.0214142.s002.pdf]

**Supporting information 2: Interview guide – Health workers**

1. What service do you provide? How do you treat patients? How often? What support do you provide? How effective do you think this support is? What works/does not work? Do you have any way to measure how effective the support you deliver is?

IF NO SUPPORT GIVEN: How do you think support could be provided to your patients and others in the community to prevent diabetes and improve dietary behaviour?

2. Where you get guidelines from on how to deliver your service?

   Probes: How are your resources provided/decided upon (e.g. District hospitals, government departments, how is it decided how many medicines and resources are available to you to give to patients etc.?)

3. Can you describe the patients that you see?

   Probes:

   - Age, socio economic group, gender, ethnicity
   - Proportion of patients with diabetes/high blood glucose levels and severity of patients presenting with diabetes/high blood glucose levels
   - Proportion of overweight/underweight patients
   - NON HOSPITAL ONLY: what capacity is there here to measure blood glucose levels?

4. I would like to ask you how you find talking to your patients. Which patients do you find it easier/more difficult to talk to? Which patients are the most receptive to treatment? How does the age, gender or ethnicity/religion or socio-economic group of patients effect how you relate to them?
5. I would like to ask you about the dietary patterns you observe amongst your patients. Is there a common pattern of dietary behaviour you observe BEFORE diagnosis and AFTER diagnosis? Do you see any changes in dietary behaviour after diagnosis?
6. [Capacity] I would like to ask you about how aware you think your patients are of the importance of a healthy diet to their health. Can you tell me to what extent you think your patients understand the causes and consequences of diabetes/high blood glucose levels?

   Probe: To what extent have you observed differences between men/women's ability to eat more healthily? To what extent are there differences between how poorer/better off patients control what they eat? How do you think the age of patients affects their ability to change what they eat?

7. What factors make it difficult for patients to eat more healthily? What enables them to eat more healthily?

    Probe: Religion/culture? Social pressures/support? Eating alone?

8. [Motivation] I would like to ask you about what could encourage your patients to eat more healthily. How do you think your patients could be encouraged to eat more healthily?
    Probe: Differences in motivation based on age/religion/gender
    Do family/friends encourage/discourage patients to eat more healthily?
9. [Capacity] I would like to ask you how you think you could provide better support for your patients. How do you think long term support could be provided to your patients and others in the community? Could this support be tailored in any way (men/women, old/young, religion/ethnicity)?

10. [Opportunity] I would like to ask you how the service you deliver here could be expanded into communities. Do you know of any good examples of successful services delivered to patients in communities? [ask to expand]

11. [Motivation] I would like to ask you about some specific ideas about improving dietary support for your patients, please could you say whether you think the ideas would work or not, and why:

- How likely would your patients be to attend a community event where you could learn more about healthy eating? Why?
- How likely would your patients be to attend nutrition education classes at hospital once per month? If not at hospital, would you be likely to attend classes if they were held in your community? Why/why not? Which would be easiest? [5.3 Information about social and environmental consequences, 11.2 Reduce Negative emotions]
- How willing would your patients be to take their partner or a family member to nutrition education events to learn how to eat more healthily? [3.3 Social Support (emotional)
- How likely would your patients be to listen to advice from a family member or friend who had had training on how to eat healthily?
- How willing would your patients be in taking part in a program which allows their eating habits to be recorded (e.g. once per month or week) in order to help you eat more healthily? [2.4/2.3 Self monitoring of behaviour]
- How likely would your patients be to take a lunchbox into work so they can eat a meal during the day? Why/why not? [1.4 Action Planning]

Probe: What issues would exist if your patients took a lunchbox into work? Social stigma about different types of food?

- How helpful would your patients would find a reminder to eat certain foods daily or weekly help you to improve their dietary behaviour? [1.4 Action planning, 7.1 Prompts and Cues]

  Probe for whether the participant has a mobile phone and would respond well to a text message reminder to eat healthily

12. [Opportunity] I would like to ask you what affects your ability to provide more support for healthy eating to your patients. What, if any, aspects of the health service here would make it difficult/easy to provide high quality care to your patients?

    Probe: What resources would you need to increase support for your patients?
